# Supplementary material for: Sugar-sweetened beverage intake and convenience store shopping as mediators of the food insecurity–Tooth decay relationship among low-income children in Washington state
Source: PLoS One. 2023 Sep 12;18(9):e0290287. doi: 10.1371/journal.pone.0290287 (PMC10497152; doi:10.1371/journal.pone.0290287)
Supplement: S1 File — (DOCX) [file pone.0290287.s002.docx]

**Supplementary File 1. Confounder Selection**


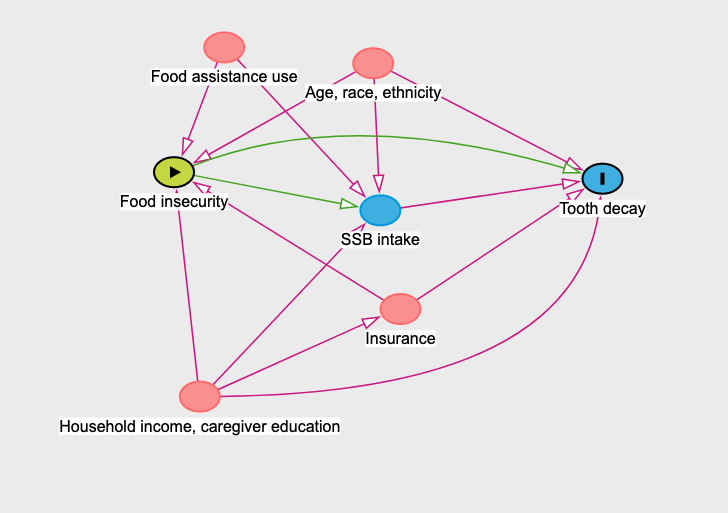


**Supplementary Figure 1. Directed acyclic graph (DAG) for food insecurity, SSB intake, and tooth decay.** The minimum set of confounders included child age, child race, child ethnicity, child insurance type, caregiver education, annual household income, and food assistance use.


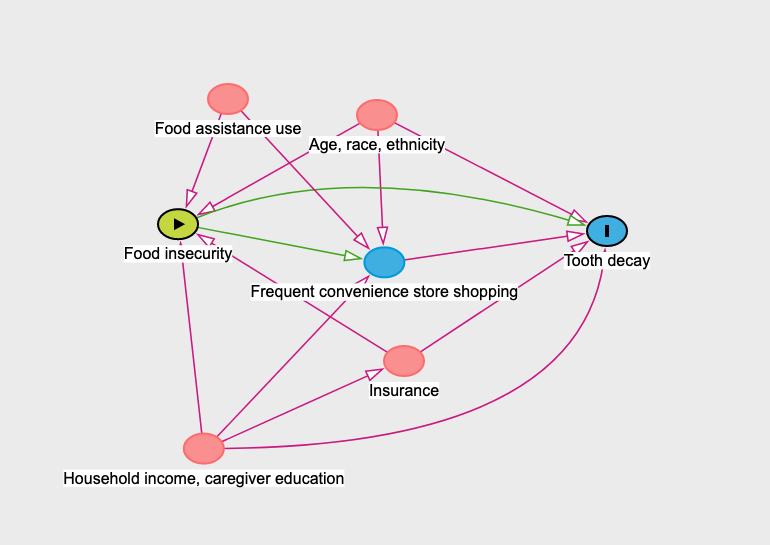


**Supplementary Figure 2. Directed acyclic graph (DAG) for food insecurity, frequent convenience store shopping, and tooth decay.** The minimum set of confounders included child age, child race, child ethnicity, child insurance type, caregiver education, annual household income, and food assistance use.
