# Supplementary material for: Sugar-sweetened beverage intake and convenience store shopping as mediators of the food insecurity–Tooth decay relationship among low-income children in Washington state
Source: PLoS One. 2023 Sep 12;18(9):e0290287. doi: 10.1371/journal.pone.0290287 (PMC10497152; doi:10.1371/journal.pone.0290287)
Supplement: S2 File — (DOCX) [file pone.0290287.s003.docx]

**Supplementary File 2. Mediation models**

**Model specification for SSB as mediator**

1) Mediator Model: $E\left[ M|X, C \right]=\beta_{0}+\beta_{1}*X+\beta_{2}*C$

2) Outcome Model: $\log\left[ [E\left[ Y|X,M,C \right] \right]=\beta_{0}+\beta_{1}*X+\beta_{2}*M+\beta_{3}*C$

Where M is natural log-transformed SSB intake, X is household food insecurity, Y is number of decayed tooth surfaces, and C represents confounders (child age, child race, child ethnicity, child insurance type, caregiver education, annual household income, and food assistance use).

**Model specification for frequent convenience store shopping as mediator**

1) Mediator Model: $\log\left[ [E\left[ M|X, C \right] \right]=\beta_{0}+\beta_{1}*X+\beta_{2}*C$

2) Outcome Model: $\log\left[ [E\left[ Y|X, M,C \right] \right]=\beta_{0}+\beta_{1}*X+\beta_{2}*M+\beta_{3}*C$

Where M is frequent convenience store shopping, X is household food insecurity, Y is number of decayed tooth surfaces, and C represents confounders (child age, child race, child ethnicity, child insurance type, caregiver education, annual household income, and food assistance use).
