# Supplementary material for: Sugar-sweetened beverage intake and convenience store shopping as mediators of the food insecurity–Tooth decay relationship among low-income children in Washington state
Source: PLoS One. 2023 Sep 12;18(9):e0290287. doi: 10.1371/journal.pone.0290287 (PMC10497152; doi:10.1371/journal.pone.0290287)
Supplement: S1 Table — (DOCX) [file pone.0290287.s004.docx]

**Supplementary Table 1. Mediating effects of SSB intake and frequent convenience store shopping in the household food insecurity–tooth decay relationship allowing for an interaction between food insecurity and mediator, for children, aged 5 to 16 years, in Seattle and South King County, 2018**

| **Mediator** | **Decayed tooth surfaces** | | | | | | |
| --- | --- | --- | --- | --- | --- | --- | --- |
|  | **Total Effect**^1^  **(95% CI)** | **p** | **Natural indirect effect**^2^  **(95% CI)** | **p** | **Natural direct effect**^3^  **(95% CI)** | **p** | **% Mediated** |
| **Log-transformed SSB intake, (fl oz/day)**^4^ | 0.43 (-0.21, 1.11) | .16 |  |  |  |  |  |
| Unexposed |  |  | 0.08 (-0.09, 0.32) | .36 | 0.38 (-0.23, 1.01) | .20 | 12.2% |
| Exposed |  |  | 0.05 (-0.13, 0.26) | .59 | 0.35 (-0.34, 1.02) | .27 | 9.2% |
| **Frequent convenience store shopping**^5^ | -3.63 (-6.25, 1.53) | .52 |  |  |  |  |  |
| Unexposed |  |  | 0.71 (-2.81, 21.95) | .37 | -3.81 (-6.35, 0.95) | .64 | 32.0% |
| Exposed |  |  | 0.18 (-0.20, 1.02) | .32 | -4.34 (-23.61, 1.11) | .92 | 17.1% |

SSB, sugar-sweetened beverage; CI, confidence interval, fl oz, fluid ounces.

^1^ The total effect can be interpreted as the differences in the average number of decayed tooth surfaces between children in food-secure and food-insecure households.

^2^ The natural indirect effect can be interpreted as the effect of food insecurity that operates through the mediator.

^3^ The natural direct effect can be interpreted as the effect of food insecurity on tooth decay that does not operate through the mediator.

^4^ SSB intake is a continuous variable measured via a 20-item beverage questionnaire. For this analysis, it was transformed as natural log(x+1) to account for right skew and to include participants who reported 0 fl oz of SSB intake.

^5^ Frequent convenience store shopping was defined as ≥2 times/week.

Poisson regression was used to model number of decayed tooth surfaces and frequent convenience store shopping as outcomes and linear regression was used to model SSB intake as an outcome. Estimates were adjusted for child age, child race, child Hispanic ethnicity, child insurance, caregiver education, annual household income, food assistance use, and number of tooth surfaces was included as a covariate in the regression models. (when tooth decay was the outcome). An interaction between food insecurity and each mediator was included in the model in order to assess the mediation estimates at each level of the exposure. All estimates for the mediation analyses are reported on the additive scale (mean differences in the number of decayed tooth surfaces) at the mean level of confounders, or the most frequent level for categorical confounders. Robust standard errors were used to generate confidence intervals.
