# Supplementary material for: Sugar-sweetened beverage intake and convenience store shopping as mediators of the food insecurity–Tooth decay relationship among low-income children in Washington state
Source: PLoS One. 2023 Sep 12;18(9):e0290287. doi: 10.1371/journal.pone.0290287 (PMC10497152; doi:10.1371/journal.pone.0290287)
Supplement: S2 Table — (DOCX) [file pone.0290287.s005.docx]

**Supplementary Table 2. Number of DMFS on household food insecurity, SSB intake, and frequent convenience store shopping among children, aged 5 to 16 years, in Seattle and South King County, 2018**

|  | **DMFS**  **(mean)** | **Mean ratio^1^ (95% CI)** | **p** |
| --- | --- | --- | --- |
| **Food insecurity** |  |  |  |
| Food-insecure | 10.4 (9.7) | Ref |  |
| Food-secure | 10.4 (10.9) | 0.96 (0.77, 1.21) | .76 |
| **Log-transformed SSB intake (fl oz/day) ^2^** | **-** | 1.03 (0.93, 1.13) | .57 |
| **SSB intake category** |  |  |  |
| 0 fl oz/day | 9.0 (9.4) | Ref |  |
| >0 fl oz/day to <8 fl oz/day | 10.6 (9.7) | 0.98 (0.64, 1.49) | .92 |
| 8 fl oz/day to <16 flo oz/day | 10.9 (10.9) | 1.10 (0.71, 1.72) | .66 |
| >16 fl oz/day | 9.9 (10.8) | 1.01 (0.63, 1.61) | .96 |
| **Any SSB intake (>0 fl oz/day)** |  |  |  |
| No | 9.0 (9.4) | Ref |  |
| Yes | 10.5 (10.3) | 1.02 (0.68, 1.53) | .92 |
| **Frequency of convenience store shopping** |  |  |  |
| Never or <1 time/month | 10.3 (8.8) | Ref |  |
| 1 time/month to 1 time/week | 10.8 (11.7) | 0.98 (0.77, 1.23) | .85 |
| ≥2 times/week | 10.0 (10.3) | 0.91 (0.67, 1.23) | .53 |
| **Frequent convenience store shopping** |  |  |  |
| <2 times/week | 10.5 (10.2) | Ref |  |
| ≥2 times/week | 10.0 (10.3) | 0.92 (0.69, 1.22) | 0.56 |

DMFS, decayed, missing, and filled tooth surfaces; SSB, sugar-sweetened beverage; CI, confidence interval, fl oz, fluid ounces.

^1^ Poisson regression with robust standard errors was used to estimate the mean ratios, 95% CI, and p-values. The mean ratio can be interpreted as the ratio of the average of number of DMFS between two groups. Models were adjusted for child age, child race, child Hispanic ethnicity, child insurance type, caregiver education, annual household income, food assistance use, and number of tooth surfaces was included as a covariate in the regression models.

^2^ SSB intake was measured via a 20-item beverage questionnaire. For this analysis, it was transformed as natural log(x+1) to account for right skew and to include participants who reported 0 fl oz of SSB intake.
