# Supplementary material for: Sugar-sweetened beverage intake and convenience store shopping as mediators of the food insecurity–Tooth decay relationship among low-income children in Washington state
Source: PLoS One. 2023 Sep 12;18(9):e0290287. doi: 10.1371/journal.pone.0290287 (PMC10497152; doi:10.1371/journal.pone.0290287)
Supplement: S3 Table — (DOCX) [file pone.0290287.s006.docx]

**Supplementary Table 3. Mediating effects of SSB intake and frequent convenience store shopping in the household food insecurity–DMFS relationship for children, aged 5 to 16 years, in Seattle and South King County, 2018**

| **Proposed Mediator** | **DMFS** | | | | | | |
| --- | --- | --- | --- | --- | --- | --- | --- |
|  | **Total Effect**^1^  **(95% CI)** | **p** | **Natural indirect effect**^2^  **(95% CI)** | **p** | **Natural direct effect**^3^  **(95% CI)** | **p** | **% Mediated** |
| **Log-transformed SSB intake, (fl oz/day)**^4^ | -0.40 (-2.86, 2.11) | .78 | 0.25 (-0.23, 0.82) | .31 | -0.65 (-3.34, 1.90) | .63 | 8.5% |
| **Any SSB intake (>0 fl oz/day)** | -0.38 (-2.87, 2.10) | .79 | 0.08 (-0.23, 0.47) | .60 | -0.47 (-3.04, 1.94) | .73 | 1.6% |
| **Frequent convenience store shopping**^5^ | -0.36 (-2.87, 2.11) | .79 | -0.03 (-0.41, 0.28) | .79 | -0.33 (-2.80, 2.09) | .82 | 0.8% |

DMFS, decayed, missing, and filled tooth surfaces; SSB, sugar-sweetened beverage; CI, confidence interval, fl oz, fluid ounces.

^1^ The total effect can be interpreted as the differences in the average number of DMFS between children in food-secure and food-insecure households.

^2^ The natural indirect effect can be interpreted as the impact of food insecurity on the number of DMFS that operates through the mediator.

^3^ The natural direct effect can be interpreted as the impact of food insecurity on the number of DMFS that does not operate through the mediator.

^4^ SSB intake is a continuous variable measured via a 20-item beverage questionnaire. For this analysis, it was transformed as natural log(x+1) to account for right skew and to include participants who reported 0 fl oz of SSB intake.

^5^ Frequent convenience store shopping was defined as ≥2 times/week.

Poisson regression was used to model DMFS and frequent convenience store shopping as outcomes and linear regression was used to model SSB intake as an outcome. Estimates were adjusted for child age, child race, child Hispanic ethnicity, child insurance, caregiver education, annual household income, food assistance use, and number of tooth surfaces was included as a covariate in the regression models (when DMFS was the outcome). All estimated estimates for the mediation analyses are reported on the additive scale (mean differences in the number of DMFS) at the mean level of confounders, or the most frequent level for categorical confounders. Robust standard errors were used to generate confidence intervals.
